# Harvest is associated with the disruption of social and fine‐scale genetic structure among matrilines of a solitary large carnivore

**DOI:** 10.1111/eva.13178

**Published:** 2020-12-14

**Authors:** Shane C. Frank, Fanie Pelletier, Alexander Kopatz, Audrey Bourret, Dany Garant, Jon E. Swenson, Hans Geir Eiken, Snorre B. Hagen, Andreas Zedrosser

**Affiliations:** ^1^ Department of Natural Sciences and Environmental Health University of South‐Eastern Norway Telemark Norway; ^2^ Département de Biologie Université de Sherbrooke Sherbrooke QC Canada; ^3^ Norwegian Institute for Nature Research Trondheim Norway; ^4^ Faculty of Environmental Sciences and Natural Resource Management Norwegian University of Life Sciences Ås Norway; ^5^ Norwegian Institute of Bioeconomy Research Svanvik Norway; ^6^ Institute of Wildlife Biology and Game Management University of Natural Resources and Applied Life Sciences Vienna Austria

**Keywords:** anthropogenic, dispersal, hunting, male mating, maternal, predator, survival

## Abstract

Harvest can disrupt wildlife populations by removing adults with naturally high survival. This can reshape sociospatial structure, genetic composition, fitness, and potentially affect evolution. Genetic tools can detect changes in local, fine‐scale genetic structure (FGS) and assess the interplay between harvest‐caused social and FGS in populations. We used data on 1614 brown bears, *Ursus arctos*, genotyped with 16 microsatellites, to investigate whether harvest intensity (mean low: 0.13 from 1990 to 2005, mean high: 0.28 from 2006 to 2011) caused changes in FGS among matrilines (8 matrilines; 109 females ≥4 years of age), sex‐specific survival and putative dispersal distances, female spatial genetic autocorrelation, matriline persistence, and male mating patterns. Increased harvest decreased FGS of matrilines. Female dispersal distances decreased, and male reproductive success was redistributed more evenly. Adult males had lower survival during high harvest, suggesting that higher male turnover caused this redistribution and helped explain decreased structure among matrilines, despite shorter female dispersal distances. Adult female survival and survival probability of both mother and daughter were lower during high harvest, indicating that matriline persistence was also lower. Our findings indicate a crucial role of regulated harvest in shaping populations, decreasing differences among “groups,” even for solitary‐living species, and potentially altering the evolutionary trajectory of wild populations.

## 1 INTRODUCTION

Anthropogenic effects on ecosystems are widespread (Vitousek, 1997). Harvest, the offtake of wildlife by humans, is pervasive and critical to many societies, for example, for food acquisition and population management. Advances in our understanding of harvest effects have generally improved its efficacy as a management tool (Linnell et al., 2010). Moreover, there are a clear differences on the impact from humans and natural predators on vital rates, which could be important for understanding harvest effects (Zeckhauser, 2017). Relative to natural predation, human harvest reduces lifespans of individuals that might otherwise experience little predation and high survival and can disrupt populations when selective or excessive (Darimont et al., 2009; Zeckhauser, 2017). Selective harvest can select for specific phenotypes (Leclerc et al., 2019; Milner et al., 2007) and alter stage age distributions, which can, in turn, influence population growth (Pelletier et al., 2012) and evolution (Allendorf et al., 2008). Intensive harvest can have long‐lasting population effects (Allendorf & Hard, 2009), favor traits that enable individuals to “cope” with human‐induced mortality, and increase their survival probability (e.g., Lone et al., 2015, Van de Walle et al., 2018), but affect “wild” traits (sensu Mysterud, 2010) that could enhance long‐term population persistence (Allendorf et al., 2008).

Harvest can also disrupt population social structure (e.g., Esteban et al., 2015; Little et al., 1993), which is an important driver of genetic structure (e.g., Storz, 1999), by (a) removing adult females in matrilines and adult males that disproportionately contribute to reproduction (Archie et al., 2008; Turner et al., 2016) and/or (b) by altering spatial demography, including sex‐specific natal dispersal and the distribution of mating success (Dieckmann et al., 1999; Ferreira da Silva et al., 2014; Harris et al., 2002). These disruptions affect gene flow, effective population size, and extinction risk (Kuparinen et al., 2016; Rick et al., 2017; Vucetich et al., 1997).

Social structure, spatial demographic patterns, and recruitment are crucial for fine‐scale genetic structure (FGS) caused by the nonrandom distribution of genotypes (Parreira & Chikhi, 2015; Storz, 1999). A disrupted social structure and spatial demographic patterns can affect the genetic make‐up of groups, suggesting long‐term effects of harvest on populations. For example, harvesting wolves (*Canis lupus*) on the periphery of their territories in Algonquin Park, Canada, reduced kin‐based composition of packs and possibly affected evolutionarily important social patterns (Rutledge et al., 2010).

Despite these potential genetic effects of harvest, studies have rarely tracked temporal changes in harvest intensity, spatial demography, and FGS (Harris et al., 2002, but see Naude et al., 2020). Changes in the FGS of harvested populations can be identified by interlinking sociospatial and genetic information (a pedigree). Assessing both may allow early detection of an altered social structure and evolutionary trajectory.

Many social mammals form matrilineal groups, that is, individuals with common female ancestors (Clutton‐Brock, 1989, 2009). Females in solitary species often cluster spatially into matrilines, due to female philopatry (Greenwood, 1980), which influences FGS (e.g., Kappeler et al., 2002; Ratnayeke et al., 2002). Differences in reproductive success (Rosenbaum et al., 2002) and male mating patterns among matrilines can also affect FGS (Buchalski et al., 2014). For example, the poaching of dominant male African elephants (*Loxodonta africana*) that randomly mate among matriarchal social groups likely decreases FGS (Archie et al., 2008). Although demographic patterns are typically more immediately important for populations than limited genetic variation, we must understand how demographic and genetic factors interact (Lande, 1988).

Here we evaluate whether harvest intensity affected the social and genetic structure in a brown bear (*Ursus arctos*) population in southcentral Sweden from 1990 to 2011. Although brown bears are considered solitary (Craighead et al., 1995), females exhibit matriline‐based spatial clusters (Støen et al., 2005). Dispersal is inversely density‐dependent (Støen, Zedrosser, Saebo et al., 2006), and bears move to nearby spatial vacancies created by harvest (Frank, Leclerc et al., 2017). We used 30 years of individual‐based genetic and demographic data to track changes in family relationships and FGS. We predicted that (a) high harvest intensity decreases FGS, that is, the distribution of genotypes becomes more random, among females and matrilines; (b) survival for both sexes is lower for all ages during high harvest, particularly for adults (≥4 years); (c) lower survival of mother–daughter pairs during high harvest; (d) an increase in the proportion of unique males siring offspring during high harvest; (e) changes in FGS are reflected in spatial configuration of related individuals on the landscape, that is, less clustering or weaker negative relationship between pairwise distances of home ranges for female kin during the high harvest; and (f) dispersal distances are shorter for both sexes during high harvest, as dispersing bears occupy nearby harvest‐induced vacancies.

## 2 MATERIALS AND METHODS

### 2.1 Study area and population

The study area was ~160,000 km^2^ intensively managed forest in southcentral Sweden (61°N, 15°E). The Scandinavian brown bear population increased from near extirpation in 1930 to ~3000 individuals today (Kindberg et al., 2011; Swenson et al., 1994). Our study population represents the southernmost subpopulation in Scandinavia (Manel et al., 2004). Population density averages ~30 bears/1000 km^2^ (Solberg et al., 2006). We captured 456 bears from a helicopter from 1985 to 2014 or roughly 50%–80% of the population in southern core (Bellemain et al., 2005b; Zedrosser et al., 2006), determined the sex and the ages of individuals not captured as yearlings from a vestigial first premolar (Matson, 1993), and took tissue and hair samples for DNA analyses. Bears were fitted with VHF (1985–2014) and GPS collars (since 2003; GPS Plus; Vectronic Aerospace GmbH Berlin, Germany), with varying location fix schedules (once a week for VHF, ≤1 hr for GPS). See Arnemo and Evans (2017) for capture details, which followed a protocol approved by the Swedish Board of Agriculture, Uppsala Ethical Committee on Animal Experiments, the Swedish Environmental Protection Agency, and the Norwegian Food Safety Authority.

### 2.2 Hunting regime and harvest intensity periods

In Sweden, bear hunting generally starts 21 August and lasts until the annual quota is filled (Swenson et al., 2017). Approximately 7% (range: 4%–10%) of this population has been harvested annually since 1942 (Swenson et al., 2017). Harvest intensity (proportion of marked bears killed by hunters) in the study population was low (mean: 0.13) during 1990–2005 and high (mean: 0.28) during 2006–2011 (Gosselin et al., 2015, Van de Walle et al., 2018), which corresponds to the first observed decrease in population size (Figure S1; Swenson et al., 2017). Harvest selectivity of sex and age is low; the sex ratio (M:F) of the harvest was ~1:1 (Bischof et al., 2009).

### 2.3 Genetic methods

DNA sources were tissue and hair from both captured and dead bears from 1990 to 2014 (Figure S2); dead bears were mostly hunter‐killed (79%). Every dead bear must be examined by the Swedish State Veterinary Institute, and tissue samples are taken for analyses. Tissue was stored in 95% alcohol prior to DNA extraction (Bellemain et al., 2005). Hair was stored dry in paper envelopes. We used samples obtained from individuals with multiple captures and/or dead recoveries to assess genotyping error rate. The genotyping of 16 microsatellites loci (Table S1) was performed in the Laboratory of Alpine Ecology (LECA) and the Norwegian Institute of Bioeconomy Research (NIBIO), following the protocol from Waits et al. (2000) and a modified protocol of Taberlet et al. (1997) and Andreassen et al. (2012), respectively. Genotyping efforts were calibrated between the laboratories (Aarnes et al., 2009), resulting in 1614 individual genotypes. Error rates were calculated using 120 individuals that were genotyped twice (Table S1).

### 2.4 Pedigree construction

We used Cervus 3.0 (Kalinowski et al., 2007) for parentage assignment and pedigree construction. Assignments of a father (with a “known mother” from field observations, *N* = 321) or both parents (*N* = 1142) were based on the relative log‐likelihood of each assignment (∆LOD). Critical ∆LOD scores with a 95% confidence level were assessed by simulations, and candidate parents were determined based on minimum ages of first reproduction (males = 3 years, females = 4 years; Støen, Zedrosser, Wegge et al., 2006; Zedrosser et al., 2007). We used COLONY (Jones & Wang, 2010) for sibship reconstruction (settings in Table S2), which simultaneously reconstructs unknown father genotypes, enabling recovery of potential fathers and sibship (95% confidence) missed in Cervus's parentage assignment. Sibship reconstruction was assessed for individuals with known mothers, but without assigned fathers (*N* = 68).

### 2.5 Population and matriline datasets

Only adult (≥4 years) females alive from 1990 to 2011 were included in the “population” (*N* = 337) and “matriline” (*N* = 109) datasets for FGS analyses. Each bear from each dataset was categorized into either “low” (1990–2005) or “high” harvest (2006–2011), depending on which “period” it had spent most of its life, based on age at and year of death. The matriline dataset was a subset of the population dataset, based on matriline assignation, that is, common female ancestry; each bear was given a “matriline ID” derived from the pedigree. Due to likely missing maternal links in the pedigree and to avoid falsely identifying founding matriarchs, we counted the number of living adult females of matrilines across time (Figure S3). False matrilines or those missing important maternal links would likely have a fewer maximum number living adult females during its tenure. We set a threshold for the minimum number of adult females that must have lived during at least one year of a matriline's tenure to be considered “true” and calculated the number of resulting matrilines. The number of resulting matrilines was plotted against an increasing threshold (from 1 to 10 adult females), and the final chosen threshold was where the curve flattened out, that is, four adult females yielding eight “true” matrilines (Figure S4). The survival analysis dataset was based on reconstructed lifespans of marked bears. FGS metrics were calculated on both population and matriline datasets, either using the variable "period" or "matriline ID" as the population subdividing unit (see below). For more details on reconstructing lifespans and matriline assignment, see Appendix S1.

### 2.6 Pairwise individual relatedness estimates

The pedigree and microsatellite data were used to calculate pairwise relatedness values, with the latter implementing Lynch and Ritland's method (hereafter LR; Lynch & Ritland, 1999). Pedigree‐derived and LR estimates used together can validate one another and increase confidence in the results (Wang, 2016). We considered Spearman's correlation coefficients ≥0.25 as within normal ranges of relatedness estimates for the matriline dataset (Csillery et al., 2006).

### 2.7 Fine‐scale genetic structure analyses

We calculated Weir and Cockerham's unbiased estimator of *F*_ST_ (Weir & Cockerham, 1984), Hedrick's *G*'_ST_ (Hedrick, 2005), Jost's *D* (Jost, 2008), and Nei's *G*_ST_ (Nei, 1973; Nei & Chesser, 1983) with the packages “hierfstat” (Goudet, 2005) and “mmod” (Winter, 2012). We first used harvest intensity (low versus high) as population subdividing units for population and matriline datasets to detect whether there was a difference in FGS among adult females between these periods. To detect whether FGS among matrilines had changed between low and high harvest, matriline ID was used to further subdivide the population (“matriline dataset”), for which all FGS metrics were independently calculated per period. All FGS metrics were bootstrapped (iterations = 1000) across all loci. We used *t* tests (*α* = 0.05) for evaluating differences between low and high harvest bootstrapped FGS values among matrilines. To test whether observed FGS values were different than produced by chance, we used permutation tests by randomly shuffling group membership of individuals, that is, which population subdivision they belonged to (“period” or “matriline”), and recalculated each FGS metric.

Because bears are long‐lived, several bears spanned both harvest periods (hereafter “straddlers”; 61%–65% of bears in both population and matriline datasets). We assigned straddlers to either period according to the period it had lived most of its life. We randomly placed bears that evenly straddled both periods into low or high harvest (sample function in base R; *N* = 19 or 6% of 337 in the population dataset; 4 or 4% of 109 in the matriline dataset). We used permutation and bootstrap calculations of FGS metrics to assess whether findings were significantly (*α* = 0.05) different from random chance and between harvest periods, respectively.

### 2.8 Distances between home range centroids and pairwise relatedness

We estimated natal HR centroids for females and males, that is, the HR centroid while with their mothers, and all available subsequent HR centroids following separation. We used the mean of all distances observed between centroids of each bear's annual HRs and its natal HR. For more details on HR centroid estimation, see Appendix S1 and Rivrud et al. (2019). We compared HRs of adult bears with their natal ranges to determine putative dispersal distances, which we compared between low and high harvest using a Mann–Whitney U test (*α* = 0.05). To assess the kin‐related spatial structure between harvest intensities, we fitted generalized additive models (GAMs) to the response variable “pairwise relatedness” and with each harvest intensity having its own smoothing function on pairwise distances among adult female HR centroids.

### 2.9 Survival probabilities

To test whether a male's or female's probability to survive to a given age was influenced by harvest period, we used a Cox proportional hazards model (Cox, 1972). We used all marked males and females and their reconstructed ages from 1990 to 2011 as the “survival time” and modeled the “event” death. Bears and their respective ages were partitioned into a covariate “period” as either “low” or “high” harvest. In quantifying the probability of joint mother–daughter survival, the event occurred when at least one individual in a mother–daughter pair died, but otherwise had the same model structure as the male and female survival analyses. We used the package “survival” and tested the proportionality of hazards assumption (*α* = 0.05) for each model (Therneau & Lumley, 2009).

### 2.10 Male mating patterns

The pedigree was used to calculate the total number offspring produced and the number of unique fathers during low and high harvest. We subset the pedigree for all offspring that were conceived during the mating year (birth year minus one year) from 1990 to 2011. We tested for changes in the ratio of total sires to total offspring between periods using a bootstrap sample (*N* = 1000, *t* test, *α* = 0.05). We used R (R Core Team, 2019) for all analyses.

## 3 RESULTS

### 3.1 Fine‐scale genetic structure and genetic diversity across harvest intensity

**FIGURE 1:**
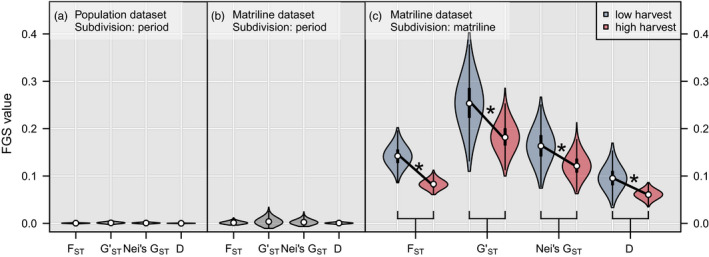
Female fine‐scale genetic structure (FGS) in brown bears between low and high harvest intensity in southcentral Sweden from 1990 to 2011 using *F*_ST_, *G*'_ST_, Jost's *D*, and Nei's *G*'_ST_. FGS metrics were calculated on a population dataset consisting of all females ≥4 years (*N* = 337) and a matriline dataset (*N* = 109) in which all females ≥4 years were assigned to a matriline. We used harvest “period” for population subdivision to assess whether females depicted FGS between low and high harvest for both population and matriline datasets (panels a and b). We used 8 unique “matriline ID’s” as the population subdivision to estimate FGS among matrilines (panel c; low: *N* = 57, high: *N* = 52 individuals). All indices were bootstrapped (iterations = 1000) across 16 microsatellite loci, and low and high harvest bootstrap values were compared using *t* tests. There was little evidence of genetic structure among females between periods (*F*_ST_ < 0.0002, *G*'_ST_ = 0.001, Jost's *D* < 0.001, Nei's *G*'_ST_ < 0.0002). However, FGS was evident among matrilines, which decreased significantly (*) from low to high harvest for each FGS metric (*F*_ST_: from 0.13 to 0.08, *p* < 0.001 ; *G*'_ST_: from 0.25 to 0.18, *p* < 0.001 ; Jost's *D*: from 0.16 to 0.12, *p* < 0.001 ; Nei's *G*'_ST_: from 0.10 to 0.06, *p* < 0.001)

There was no evidence of change in FGS for adult females between low and high harvest (Figure 1, Figure S4 and Table S3; low, *N* = 197; high, *N* = 140; FGS range: <0.001 – 0.001, all permutation tests, *p* > 0.587) or matriline datasets (Figure 1, Figure S4 and Table S3; low, *N* = 57; high, *N* = 52; FGS range* = *0.001–0.004, all permutation tests, *p* > 0.516). Thus, grouping of adult female genetic variation was similar between low and high harvest. However, we found that FGS of females among matrilines decreased from low to high harvest for each FGS metric used (Figure 1 and Table S3; all *t* tests, *p* < 0.001 for each pairwise‐period comparison), indicating that although genetic variation was similar between periods for all females, it changed among matrilines. All estimated FGS metrics among matrilines were significantly different from those produced by chance (Figure S5 and Table S3, all permutation tests, *p* < 0.001). Post hoc analyses on allelic richness showed an increase from low to high harvest, but this effect varied by locus and was dependent on the whether the matriline or population dataset was used for comparison (Figure S7). Expected heterozygosity was, however, comparable between both datasets and between low and high harvest (range: 0.632–0.661).

### 3.2 Distances between home range centroids and pairwise relatedness

**FIGURE 2:**
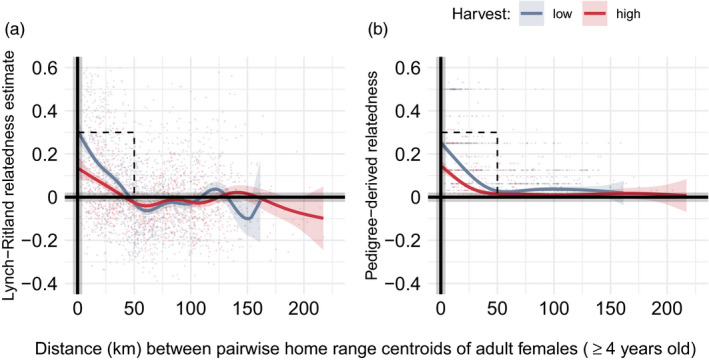
Pairwise relatedness of adult female brown bears (≥4 years) using Lynch–Ritland's estimator (LR; a) or pedigree‐derived (b) against pairwise distances (km) between home range centroids from 1990 to 2011 in southcentral Sweden. For each relatedness metric, the response pairwise relatedness and predictor pairwise distances were fitted with generalized additive models with a smoother function on pairwise distances and by each harvest period. For both metrics, the high harvest depicted lower pairwise relatedness at distances up to ~50 km (black dotted rectangle) between home range centroids (LR response: *β* = −0.029, *p* < 0.001; pedigree response: *β* = − 0.034, *p* < 0.001)

High harvest was associated with significantly lower pairwise relatedness, with decreasing distance between centroids, regardless of whether LR‐estimated or pedigree‐derived relatedness was used (Figure 2; reference = low harvest; LR response: *β* = −0.029, *SE* = 0.009, *p*‐value = <.001; pedigree response: *β* = −0.034, *SE* = 0.004, *p*‐value = <.001). Although more closely related females were spatially clustered in each harvest period, pairwise relatedness was lower for high harvest in all distances up to ~ 0 km (Figure 2).

### 3.3 Survival probabilities

**FIGURE 3:**
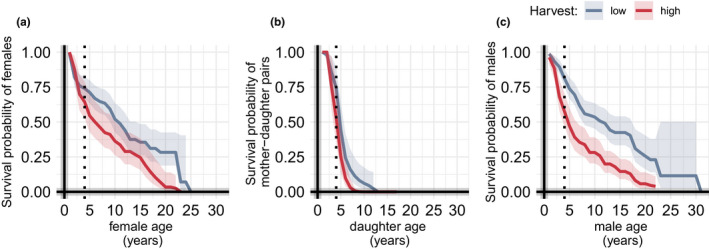
Survival probability using Cox proportional hazards model for marked brown bears in southcentral Sweden from 1990 to 2011; (a) females, (b) joint survival of mother–daughter pairs, and (c) males. Broken lines indicate age 4, that is, when a female or male was considered reproductive, and when a female was considered a part of a matriline. Matriline formation and maintenance appeared less probable during high than to low harvest. Both reproductive females and males had lower survivorship during high than low harvest, particularly after reaching adulthood (>4 years)

High harvest significantly lowered the survival of females, mother–daughter pairs, and males (Figure 3, panels a, b, and c; low harvest = reference; *β* = 0.540, *SE* = 0.183, *p* = 0.003; *β* = 0.833, *SE* = 0.187, *p* = <.001; and *β* = 0.777, *SE* = 0.162, *p* < 0.001, respectively). Adult females incurred slightly reduced probabilities of survival during high harvest; the probability for a female to survive at age 10 was 0.52 (95% CI: 0.43–0.64) and 0.36 (95% CI: 0.26–0.50) for low and high harvest, respectively (Figure 3a). Furthermore, model predictions indicated lower survival probability for mother–daughter pairs at high compared to low harvest (Figure 3b). Males had a reduced survival probability during high harvest; at age 10, male survival was 0.54 (95% CI: 0.45–0.63) during low and 0.28 (95% CI: 0.17–0.39) during high harvest (Figure 3c).

### 3.4 Sex‐specific dispersal and male mating patterns across harvest intensity

**FIGURE 4:**
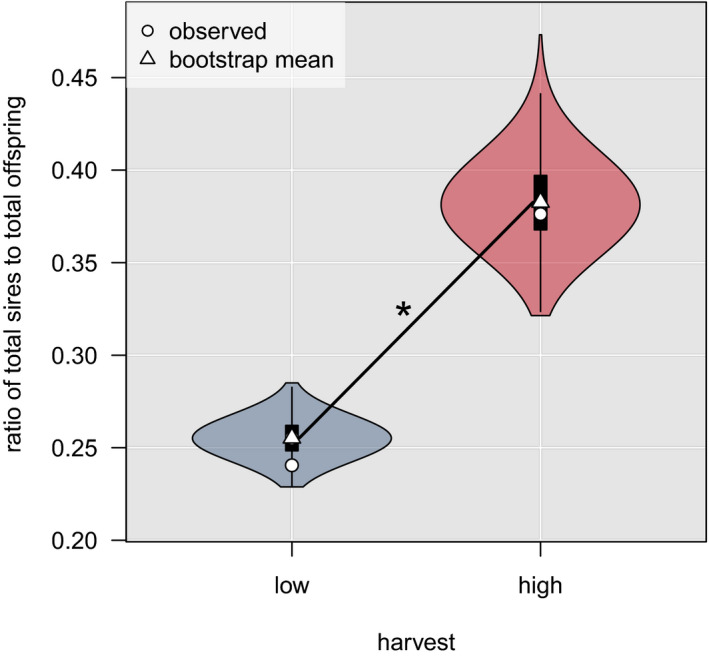
The ratio of unique male brown bear sires to total offspring (white dots) during periods of low and high harvest intensity (offspring: low = 405, high = 233; sires: low = 131, high = 112) in southcentral Sweden from 1990 to 2011. Each low‐ and high harvest dataset was bootstrapped (*N* = 1000), and ratios were recalculated. Mean bootstrap values (white triangles) were compared using a *t* test. The period for high harvest had a significantly higher (*) proportion of unique males siring offspring than low harvest (observed: 0.24–0.38, bootstrap mean: 0.26–0.38; *p* < 0.001)

Female dispersal distances decreased significantly between low (11.5 km) and high (6.5 km) harvest (Figure S6a; MWU test, *p < *0.01), whereas there was no change in male dispersal distances (Figure S6b; low = 85.0, high = 81.2 km, MWU test, *p* = 0.66). Postdispersal breeding success was distributed across significantly more males during high harvest (Figure 4, observed ratio of total fathers to total offspring: low = 0.24, high = 0.38, *t* test, *p* < 0.001).

## 4 DISCUSSION

We documented FGS, the spatial proximity of related individuals, survival rates, dispersal distances, and male mating patterns during low and high harvest intensities. As expected, the partitioning of genetic variance was weaker among matrilines under high harvest, and shorter female dispersal distances and a relative increase in successfully reproducing males helped reduce differences in genetic composition among matrilines. Although spatial genetic autocorrelation of females occurred until ~50 km at both low and high harvest, relatedness among spatially clustered pairs of females was significantly smaller during high harvest and was magnified as females became closer. This occurred despite decreased female dispersal distances during high harvest, which theoretically should lead to an increase of closely related females at shorter distances (Bohonak, 1999). Thus, increased genetic admixture from the increased number of sires probably influenced the reduction in FGS more than female dispersal distances (e.g., Chesser, 1991).

### 4.1 Harvest‐induced altered mating patterns

Skewed male reproductive success in relation to body size and age suggests male dominance structures in this population (Zedrosser et al., 2007). Shorter female dispersal distances during high harvest imply a stronger clustering of females, likely allowing males to overlap with more potential mates and exclude competing males. However, this is likely offset by harvest of females. Multiple paternity (two to three sires per litter) could explain the observed increase in number of males contributing to progeny, but the frequency of these litters has remained constant over time and across harvest periods, so it is unlikely to explain the observed decrease in FGS (S. C. Frank *unpublished*). Furthermore, male social and dominance structures appear sensitive to male turnover (Leclerc et al., 2017). Harvest‐created HR vacancies cause a spatial reorganization in both sexes by attracting same‐sex neighbors and provide space for immigrants (Frank, Leclerc et al., 2017). Harvest can increase the number of successful mating young males (Poteaux et al., 2009), thus disrupting social organizations (Lane et al., 2011; Lott, 1991). Although older and larger males have higher breeding success in polygynous species (Andersson, 1994), disruptions to male social structure do not require selective harvest (Bischof et al., 2008; Proaktor et al., 2007). Harvest can shift breeding success to males of similar age or size as the killed animals (Ishengoma et al., 2007) or it can redistribute breeding success across different ages and sizes or to more males (Moore et al., 2015; Zedrosser et al., 2007). The effect of harvest on male breeding success is likely not linear and depends on operational sex ratios (Newbolt et al., 2017). However, given a near‐equal harvest sex ratio during our study period, harvest intensity might be more critical for shifting male breeding success (Mysterud, 2011). Furthermore, harvest does not need to be selective to have genetic effects (Engen et al., 2014; Law, 2007; Rouyer et al., 2012), and decreasing harvest intensity alone helps to avoid harvest‐induced evolutionary changes (Kuparinen & Festa‐Bianchet, 2017). We suggest that high harvest (≥10%) and associated high male turnover destabilized male dominance structure in our study population (Andreassen & Gundersen, 2006; Ausband et al., 2017; Gosselin et al., 2017), which increased the number of successfully reproducing males and decreased FGS among matrilines. Similarly, the removal of adult males from matrilineal‐based groups in killer whales (*Orcinus orca*) and African elephants disrupted group cohesion and genetic composition (Archie et al., 2008; Wade et al., 2012). Bears live solitarily, except when rearing offspring, suggesting that disruptions to social structures are important across a range of sociality (Parreira & Chikhi, 2015). The higher relative mortality risk for males than females during high harvest (Figure 3) could indicate an increasingly male‐biased harvest in more recent years (Frank, Ordiz et al., 2017). As male turnover increases, relatively more males could successfully breed among matrilines, increasing their relative contribution to gene flow among matrilines, thereby reducing FGS at this scale.

### 4.2 Female survival, matriline formation, and persistence

Female reproductive lifespan is greatly reduced by harvest in this study population (Bischof et al., 2018; Zedrosser et al., 2013), with 71% of all recorded adult female mortality coming from legal harvest between 1990 and 2011. Heterogeneity in female reproductive success due to differences in lifespan could ultimately affect matrilineal genetic structure (Rosenbaum et al., 2002). Although sex and age harvest selectivity is considered low in this population (Bischof et al., 2009), high harvest appears to select for longer maternal care duration and smaller adult female size (Leclerc et al., 2016, Van de Walle et al., 2018). If such selectivity has a genetic basis and is asymmetric among matrilines, then increased FGS can arise, but FGS was lower in our study and not suggestive of this effect. In species that typically form maternal‐based social groups, high harvest can decrease FGS by disrupting their social cohesion and mating patterns (Comer et al., 2005). Solitary female bears incurred greater mortality risk during higher harvest (Van de Walle et al., 2018), and the survival of mother–daughters across time was lower during high harvest, suggesting a lower probability for the formation and maintenance of matrilines. In fact, the number of members in most matrilines either stabilized or decreased during high harvest (Figure S3). Any social or other resource‐based benefits conferred to individual fitness from philopatry and matrilineal structures are likely influenced by harvest mortality, but this is little studied in solitary species (but see Clutton‐Brock & Lukas, 2012).

Disruptions to social structures can affect mating patterns and enhance sexually selected infanticide (SSI) through increased contact between unfamiliar male and female mates during the breeding season (Gosselin et al., 2017; Leclerc et al., 2017). In response, females adapt countermeasures against SSI and promiscuously mate with several males to reduce SSI risk (Bellemain et al., 2006). This strategy should be more pronounced during high harvest (Gosselin et al., 2015, 2017), which should increase variability around population growth estimates (Gosselin et al., 2015), underscoring the need to better understand the interactions between sociality and harvest‐induced effects and how they affect heterogeneity in individual fitness and population growth.

### 4.3 Study limitations

We found that high harvest decreased FGS among matrilines, but not independent of matrilines, that is, when females were grouped by harvest period alone. This suggests that harvest‐induced changes in FGS can be cryptic and occur within ca. four generations (~20 years), based on an average age of five years for primiparity (Zedrosser et al., 2009). Although an increasing population might reduce FGS, due to immigration (Nussey et al., 2005), the number of breeding females was similar between harvest periods (matriline dataset: low, *N* = 57; high, *N* = 52) and immigration from other subpopulations was low (~one immigrant per generation; Schregel et al., 2017). Both local and regional immigrants can be attracted to harvest‐induced vacancies (Frank, Leclerc et al., 2017). Therefore, an increase in more distant immigration from neighboring subpopulations would likely be influenced by harvest. Due to low immigration rates in this population (Schregel et al., 2017), it is less likely that this would have as much of an impact on FGS as high local turnover of both males and females in this subpopulation. However, harvest intensity has been linked to differences in sex‐specific dispersal patterns in large carnivore populations (Elliot et al., 2014; Robinson et al., 2008), and quotas are commonly based on population size and growth rate (Williams et al., 2002). Therefore, the effects of harvest intensity and population size or density on social, genetic, and demographic structure of wild populations could be confounding (Allendorf et al., 2008). Nevertheless, by using several FGS metrics and spatial genetic autocorrelation, coupled with individual‐based survival probabilities, dispersal distances, and male mating patterns, our study indicates that harvest likely contributes to a reduced FGS. Despite some debate on the theoretical justification for the specific use of our chosen genetic metrics (Appendix S1), all suggested a consistent, negative change that is correlated with high harvest.

The length of our study period (22 years) could be considered short for detecting genetic effects from harvest, given that brown bear generation time can reach 10 years (Kumar et al., 2017). However, hunter‐killed bears are representative of the population (Leclerc et al., 2016), and mean age of shot female bears is ~5 years in our study population (Frank, Ordiz et al., 2017), effectively truncating many females’ reproductive lifespans (Zedrosser et al., 2013). There is strong evidence that harvest is additive in this population and increasingly so across time (Bischof et al., 2009), and the relative risk of harvest compared with natural mortality generally increases with age (Bischof et al., 2018). Human access and proximity to roads have been linked to risk of harvest for bears (Penteriani et al., 2018) and other mammals (Hill et al., 2019). Road networks can not only enhance mortality risk for mammals (Gratson & Whitman, 2000; Lamb et al., 2018; Steyaert et al., 2016) but also fragment and constrain the spatial configuration of home ranges (Bischof et al., 2017), indicating its potential influence on FGS. In our study area, road networks largely comprise those built and used for forestry practices, which in turn create habitat patches of different forest age class. Habitat fragmentation can reduce gene flow and increase genetic structure among isolated portions of a population (Keller & Largiader, 2003). If matrilines are isolated or alternatively fragmented by roads, this could affect mating patterns due to barrier effects on movement during dispersal and/or breeding. We did not, however, assess the influence of habitat fragmentation or road networks on FGS, and although their influence cannot be ruled out, human landscape features are permeable and are often crossed during bear dispersal and long‐range movement (Barton et al., 2019).

### 4.4 Harvest‐induced changes in FGS and genetic diversity

Human influence over wildlife is ubiquitous (Bernardo‐Madrid et al., 2019), and most populations are exploited (Ripple et al., 2016), emphasizing the need to understand harvest effects on population dynamics and genetics (Allendorf & Luikart, 2007). Effects from harvest are most pronounced with repeated, intensive harvest and when selection occurs in both sexes (Festa‐Bianchet & Mysterud, 2018). And, harvest intensity can outweigh selection as a driver of changes to genetic composition and/or induce adaptive responses in populations (Proaktor et al., 2007) and be detrimental to population genetics, and thus persistence, that is, decrease effective population size (Allendorf & Hard, 2009). Sudden environmental changes may threaten harvested populations and increase their extinction risk (Cameron et al., 2016). However, an increased number of sires in response to harvest may increase allelic frequencies, thus improving the adaptive potential to environmental change (Baltazar‐Soares & Eizaguirre, 2016; Garant et al., 2007). Analyses on allelic richness showed an increase from low to high harvest, but this effect varied by locus and was only apparent for the matriline dataset (Figure S7), whereas expected heterozygosity was consistently high and comparable between both datasets and between low and high harvest (range: 0.632–0.661). Therefore, there is a not clear signal on whether harvest enhanced genetic diversity in this population.

Others have reported harvest‐induced increased genetic admixture among individuals within “social” groups or demes (Ausband & Waits, 2020; Little et al., 1993), but typically in group‐living species. Following a hunting ban that ended in 1943, our study population has been hunted with increasing intensity until present (Swenson et al., 2017). A word of caution is that although a population can become adapted to high harvest intensity and optimize growth under such circumstances (Sih et al., 2011), it can also exhibit a slow recovery once harvest ceases due to human‐mediated selection of suboptimal traits in the face of natural selection (e.g., Uusi‐Heikkila et al., 2015). These long‐term effects from harvest are likely at least in part due to long‐lasting disruptions to animal social systems (Lott, 1991).

The importance of sociality and social structure for populations is gaining more attention (Parreira & Chikhi, 2015). With more males contributing to progeny, less genetic differentiation occurs among matrilines, but it is unclear whether the resulting phenotypes are best adapted for long‐term population persistence in a variable environment. High harvest intensity appears to cause or contribute to rapid and cryptic changes to FGS in populations at the deme level of a solitary species, where matrilineal structures can be used to monitor changes in mating, dispersal, and emerging spatial genetic patterns. This requires several scales of analyses and long‐term individual‐based demographic and genetic data. Such data are costly and difficult to acquire, particularly for large, solitary, and elusive wildlife (Kindberg et al., 2009). However, tracking patterns of genetic variation over different generations, for example, using noninvasive sampling, may be one of few ways to capture extinction risk for some populations (Dunham et al., 1999).

### 4.5 Concluding remarks

FGS has been the basis for designating management and conservation units (Allendorf & Luikart, 2007; Vekemans & Hardy, 2004), and Schwartz et al. (2007) strongly encouraged the monitoring of potentially negative harvest‐induced genetic changes in populations and diversity for management and conservation of harvested populations. In the northern hemisphere, many bear populations of brown bear and American black bears (*U. americanus*) are not threatened and are managed through a regulated hunt, but the situation is less certain for bear species and populations nearer to and south of the equator where they are considered at high risk of extinction (IUCN, 2020). In any case, many populations lack pre‐exploitation baseline data (Rodrigues et al., 2019), especially to the degree that modern monitoring techniques can now provide, but contemporary genetic sampling will still be useful in the future. Our findings originate from an intensively managed population, and data collected on a previously unstudied, exploited population should not be erringly considered a “true” baseline in terms of anthropogenic influence. Although this is the case and current use of such information might not appear readily pertinent, managers might consider the continuous monitoring of changes in dispersal and mating patterns over time, potentially due to harvest, for future use. Authorities might also consider adopting more systematic, annual noninvasive genetic surveys similar to Norway, that is, those which yield less uncertainty (Bischof, 2019) and can better infer harvest‐induced changes to the population via dispersal, mating, and social structure. These disruptions warrant further study in their influence on the vortex effect on extinction risk, particularly for vulnerable populations (Lacy, 2000). Our findings highlight the benefit and need for long‐term studies and monitoring of wildlife and their demography and genetics to ensure evolutionary potential and enhance population persistence (Clutton‐Brock & Sheldon, 2010; Hansen et al., 2012).

## CONFLICT OF INTEREST

The authors declare no conflicts of interest.

## Supporting information

Appendix S1

## Data Availability

R code and data used in this study are deposited in the Dryad Digital Repository (Frank et al., 2020): https://doi.org/10.5061/dryad.qrfj6q5dh
