# Supplementary material for: Harvest is associated with the disruption of social and fine‐scale genetic structure among matrilines of a solitary large carnivore
Source: Evol Appl. 2020 Dec 14;14(4):1023–35. doi: 10.1111/eva.13178 (PMC8061280; doi:10.1111/eva.13178)
Supplement: Supplementary file 1 — Appendix S1 [file EVA-14-1023-s001.docx]

## Supporting Information for

# Harvest is associated with the disruption of social and fine-scale genetic structure among matrilines of a solitary large carnivore

SC Frank, F Pelletier, A Kopatz, A Bourret, D Garant, JE Swenson, HG Eiken, SB Hagen, A Zedrosser

# Appendix S1

## Methods

#### Population dataset

Using ages of marked females (alive and recovered dead; N = 201) and unmarked females (recovered dead; N= 458) for which genetic information was available, we constructed the years in which females were alive from the earliest recorded birth year to the most recent 1963-2014. Our study period began in 1990 when DNA collection began and lasted until 2011. We utilized DNA samples and genotypes though 2014, but truncated the study period by 3 years, i.e., the mean age of female bears killed in 2014, to reduce potential bias from the reconstruction of lives from recovered dead bears, which can accumulate over time, This yielded a study period from 1990-2011, which we further divided into low (1990-2005) and high harvest (2006-2011) periods and allocated bears based on which side it had lived the majority of its life or the specific year in which the bear was alive for fine-scale genetic and survival analyses, respectively. Only marked bears were used for survival analyses, as we had a known life history for each bear. Furthermore, to examine FGS, we used females ≥ 4 years old (N = 337), to include only reproductive females. We refer to this data set as the 'population data set', as not all females were successfully assigned to mothers and constituted a larger sample size of the population than those assigned to matrilines (see below).

#### Matriline dataset

A matrilineal "founder" ID, i.e., that of the oldest known female of an identified matriline, was used as the matriline ID for all her female descendants. The pedigree was incomplete, i.e., some females were not assigned mothers, which could result in erroneously assigned matriline IDs or those erringly abbreviated or self-assigned, due to missing maternal links. To increase confidence in matrilineal assignments, we used the reconstructed lives of females and their presence over time (Fig. S2). We chose a minimum threshold for the maximum number of living females observed in a matriline across years at any given time by plotting the number of matrilines resulting from the incremental increase in the threshold. We considered the threshold at which "true matrilines" (hereafter matrilines) to occur as where the curve leveled out (Fig. S2). This strategy reduced the risk of including a ‘false’ matriline or those with missing maternal links, but may also improperly exclude matrilines with few female descendants. The chosen threshold was four individual females at age ≥ 4 years living concurrently during at least one year of a matriline's tenure (Fig. S2-S3). We subset the population data set to include only those that belonged to a matriline (hereafter 'matriline data set'). FGS metrics were calculated on both population and matriline datasets, either using the variable "period" or "matriline ID" as the population subdividing unit (see *Fine-scale genetic structure analyses* in the main text).

#### Rationale for chosen fine-scale genetic structure metrics

The FGS metrics in this study are widely-used in ecological studies, but each have their own set of assumptions and drawbacks (Jost 2008, Meirmans and Hedrick 2011). Though still widely -used, F_ST_ has been criticized for having strong dependencies on within-population genetic diversity values (Meirmans and Hedrick 2011), and Nei’s G_ST_ has been criticized for not truly measuring differentiation (e.g. Jost 2008). Therefore, we also calculated Jost’s D and Hedrick's G'_ST_. The former assesses ‘true’ genetic differentiation (Jost et al. 2018) and the latter standardizes F_ST_ according to its maximum possible value possible, given the observed genetic diversity of a population (Hedrick 2005, Meirmans and Hedrick 2011).Although there has been a more recent clarification of the distinction between assessing two types of genetic structure, i.e., fixation indices (F_ST_, Hedrick's G'_ST_, and Nei’s G_ST_) and genetic differentiation (Jost's D) by Jost et al. (2018), we employ them for a similar purpose here—to detect differences in the adult female population(s) between low and high hunting periods. As the population was growing consistently throughout low harvest (Fig. S1), it follows that ‘fixation’ of alleles likely has not occurred (the basis for fixation indices), as these measures are more sensitive to demographic rates, such as population growth (or decline), and should not be used to indirectly infer migration rates (Meirmans and Hedrick 2011, Jost et al. 2018). By using matrilines as a population subdivision in our analyses, it likely violated some assumptions of most metrics, most notably the notion of equilibrium (Meirmans and Hedrick 2011) or that which requires population stability and isolation via an island model. These assumptions, however, are rarely satisfied in practice and utility of the metrics is not lost (Jost et al. 2018). For example, F_st_ is theoretically the counterbalance between genetic drift, the random disappearance of alleles due to chance, and gene flow within a population, but the magnitude of drifting allelic frequencies is likely minimal given the timespan of our study, but high harvest can augment those effects over a longer period (Allendorf et al. 2008).

# Figures and Tables

**
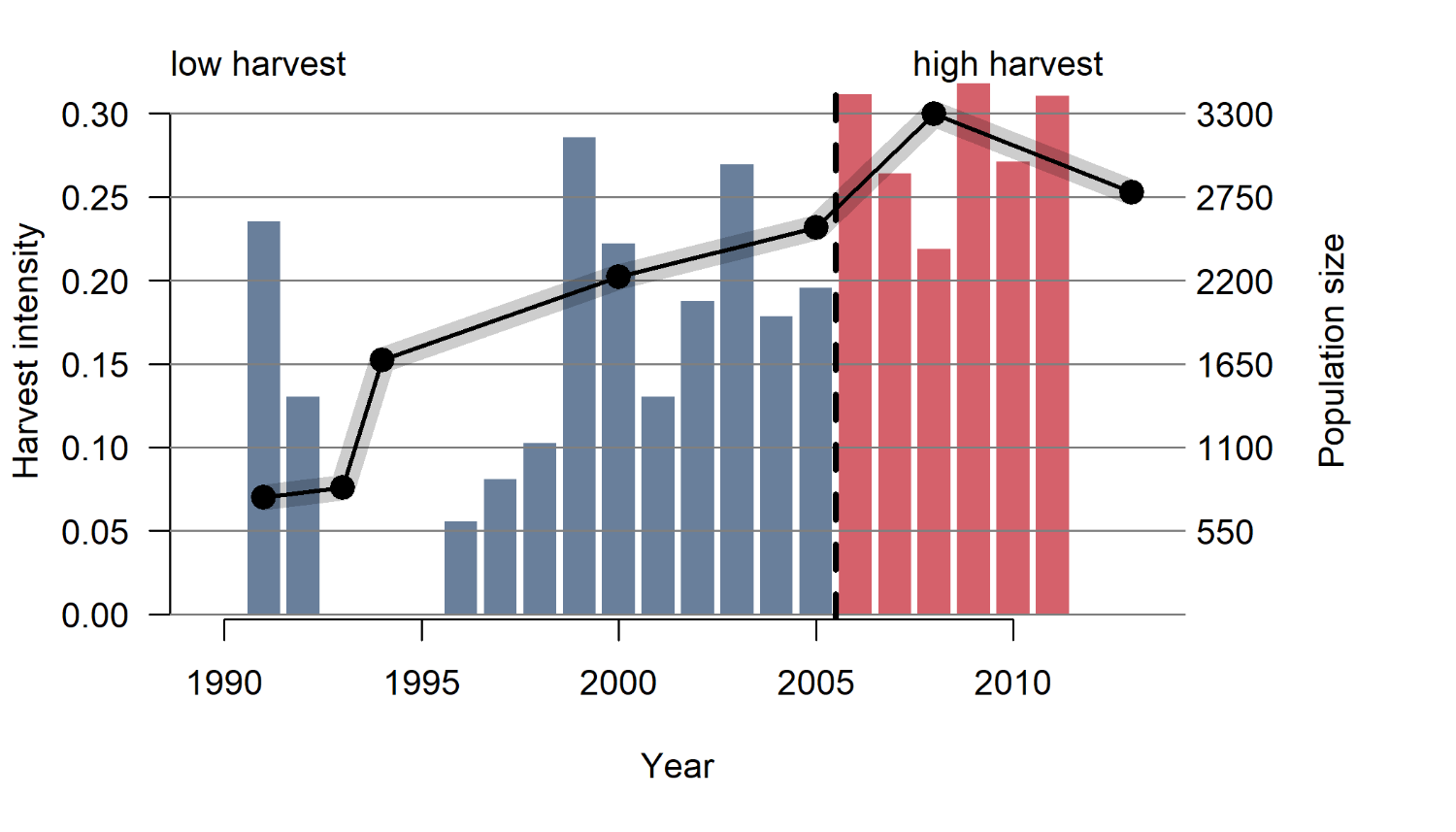
**

Fig. S1. Annual harvest intensity (vertical blue and red bars), i.e., the proportion of marked brown bears shot in Sweden, is shown in relation to annual population size (line with black dots; Swenson et al. 2017). Periods of low (1990-2005) and high harvest (2006-2011) are divided by the vertical black dashed line. Mean harvest intensity was 0.13 [range = 0 – 0.29, SD = 0.10] and 0.28 [range: 0.22 – 0.32, SD = 0.04] in low and high harvest periods, respectively.


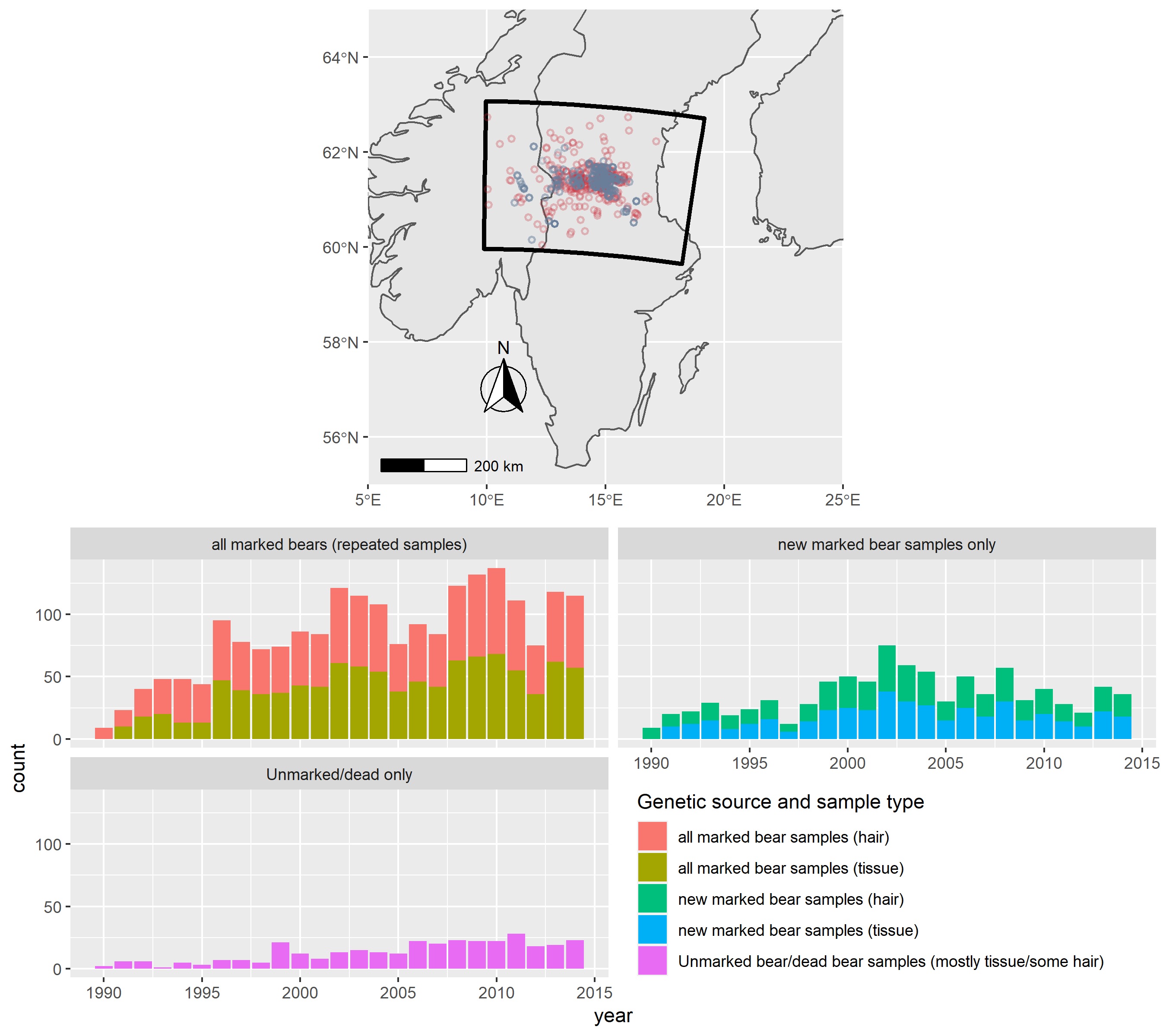


Fig. S2. Top panel: Spatial locations of genetic sources, i.e. brown bear (*Ursus arctos*) hair and tissue samples, from unmarked, dead bears (red circles) and marked bears (gray circles) in southcentral, Sweden from 1990-2014). Bottom four panels: Genetic sources (marked and unmarked bears) and sample types (hair/tissue) collected across time. Bears were sampled in repeated bouts (all marked bears in salmon and gold bars) to demonstrate sampling effort and new marked bears (blue and green bars) indicates the first time a bear was sampled. Unmarked/dead bears (purple bars) were sampled only once upon death.


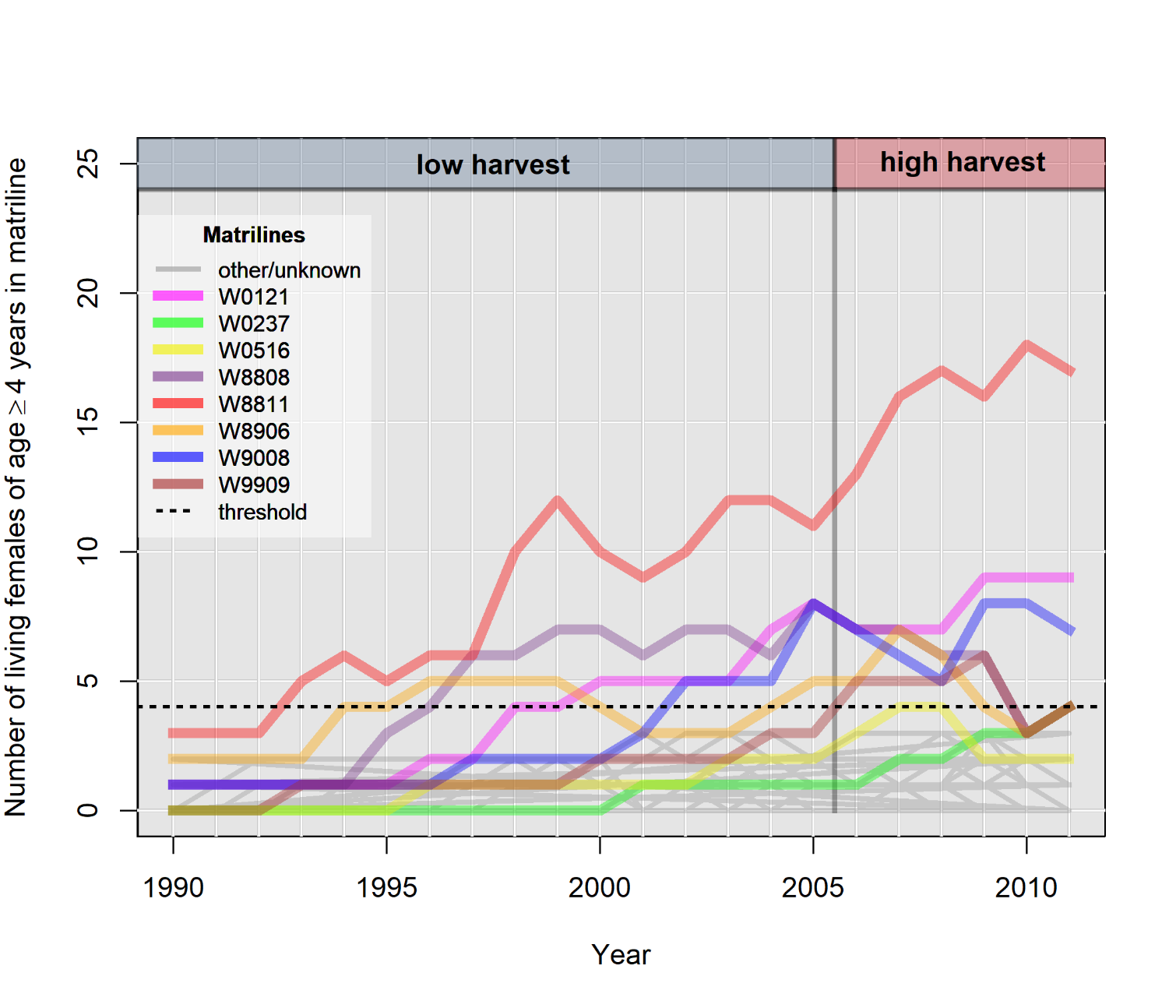


Fig. S3. The number of female brown bears belonging to matrilines in southcentral Sweden, i.e. labeled by founding females, across years from 1990-2011, grouped into periods of low (1990-2005) and high (2006-2011) hunting pressure. The horizontal broken line signifies the number of female descendants (aged ≥ 4 years) that a founding female must have to be conservatively considered a matriline in this study. The category "other/unknown" is a result of that classification, i.e., not considered a part of a matriline (see text and Fig. S2).


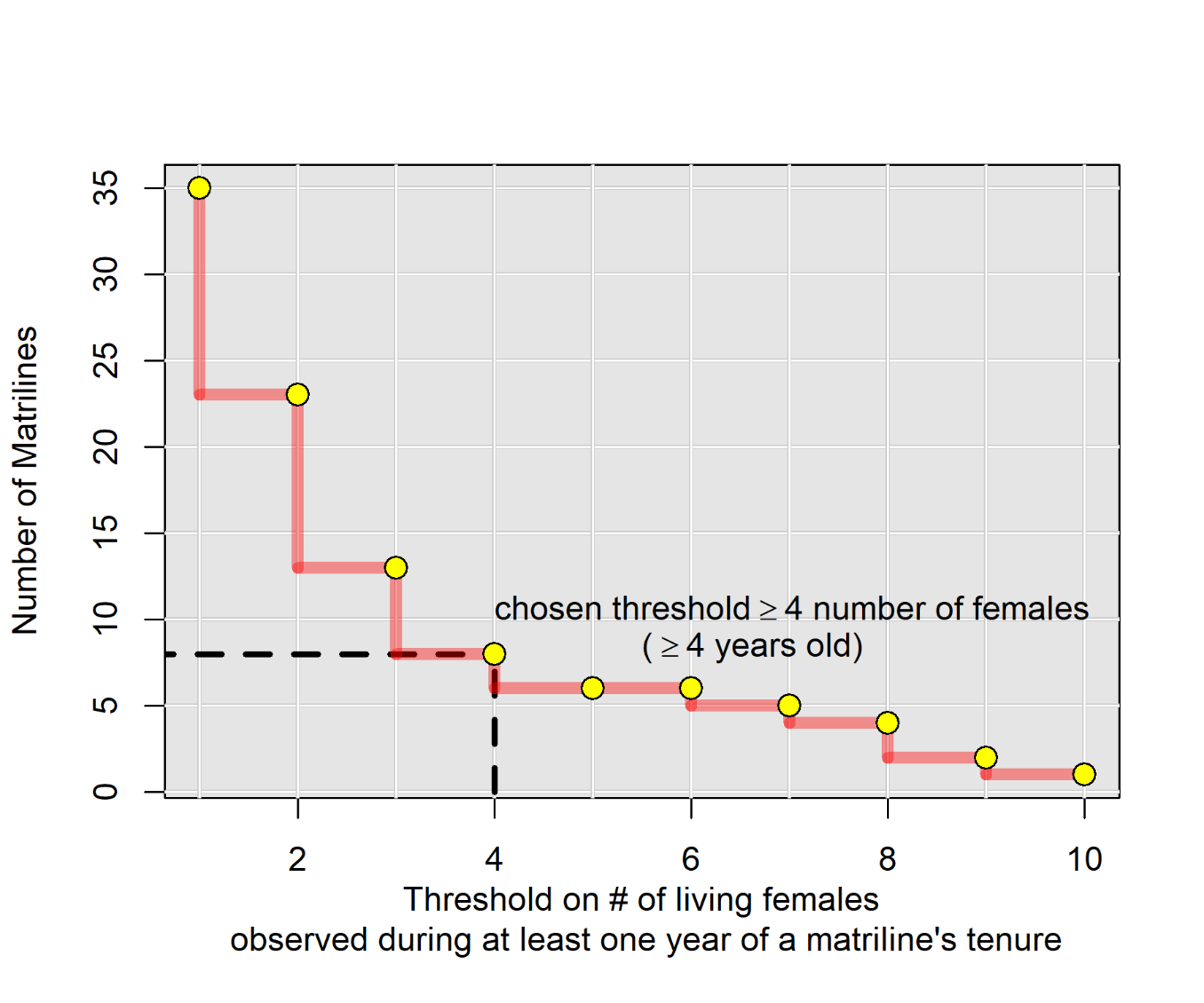


Fig. S4. The number of brown bear matrilines (y-axis) produced from variable thresholds on the minimum number of living females ≥ 4 years of age observed (x-axis) belonging to a

founding female, as depicted by yellow dots. The red steps illustrate where the decrease in number of matrilines produced by various threshold values levels out. Leveling out occurred at four living females ≥ 4 years of age, which is shown by the intersecting broken black lines. We considered 8 matrilines as 'true matrilines', i.e., those belonging to a founding female that contained four or more living females of ≥ 4 years of age during at least one year of its tenure

**
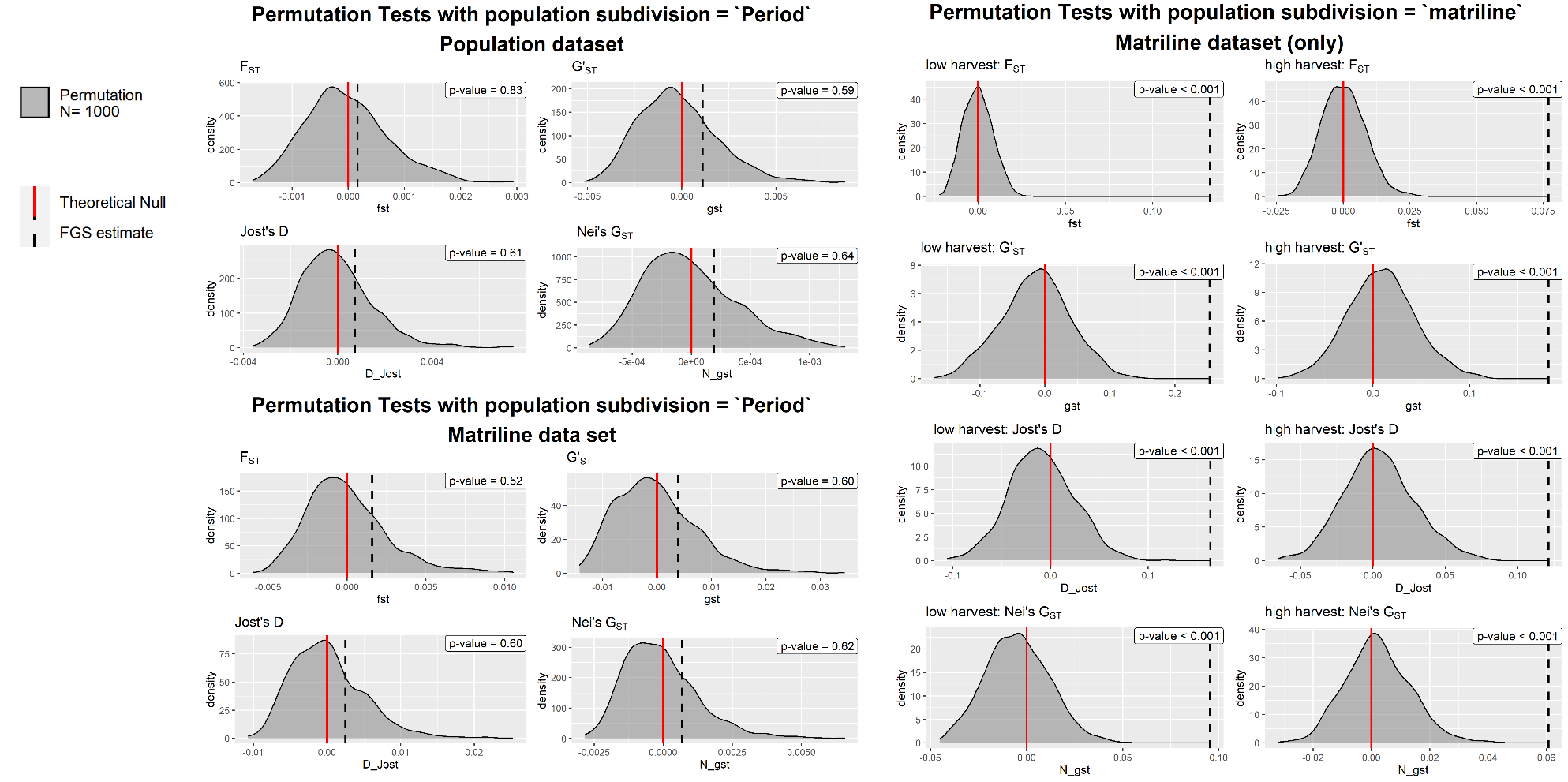
**

Fig. S5. Permutation tests (α = 0.05) for each fine-scale genetic structure (FGS) metric, i.e., Weir and Cockram's F_ST_ (Weir and Cockerham 1984), Hedrick's G'_ST_ (Hedrick 2005), Jost's D (Jost 2008), and Nei’s G_ST_ (Nei 1973, Nei and Chesser 1983) that was calculated for brown bear population (N = 337 females ≥ 4 years old) and matriline data sets (N = 109 females ≥ 4 years old) in southcentral Sweden. Table S3 shows the specific values attributed to each vertical line. The population subdivision harvest intensity period (‘period’ = the division between low and high harvest) was used with each population and matriline datasets to test whether there were structural differences between adult females (≥ 4 years old) between low and high harvest or significantly different from the null hypothesis of zero or no difference (left two columns of plots). The population subdivision ‘matriline ID’ was used to test whether matriline structures were different than otherwise produced by random (null hypothesis of FGS = zero; right two columns of plots). NB: see ‘bootstrap’ in the methods for comparing the FGS differences of matrilines between low and high hunting periods. Gray shaded areas are the distributions of permutations of a given FGS metric, theoretical null (zero) is depicted by a vertical solid red line, and the estimated FGS metric from the observed population is shown with a vertical dashed black line for a given data set. There was no evidence of genetic structure between adult females from low and high hunting periods, regardless of using either the population or matriline data sets. All observed FGS values among matrilines for each low and high hunting periods were significantly different than those produced by chance.


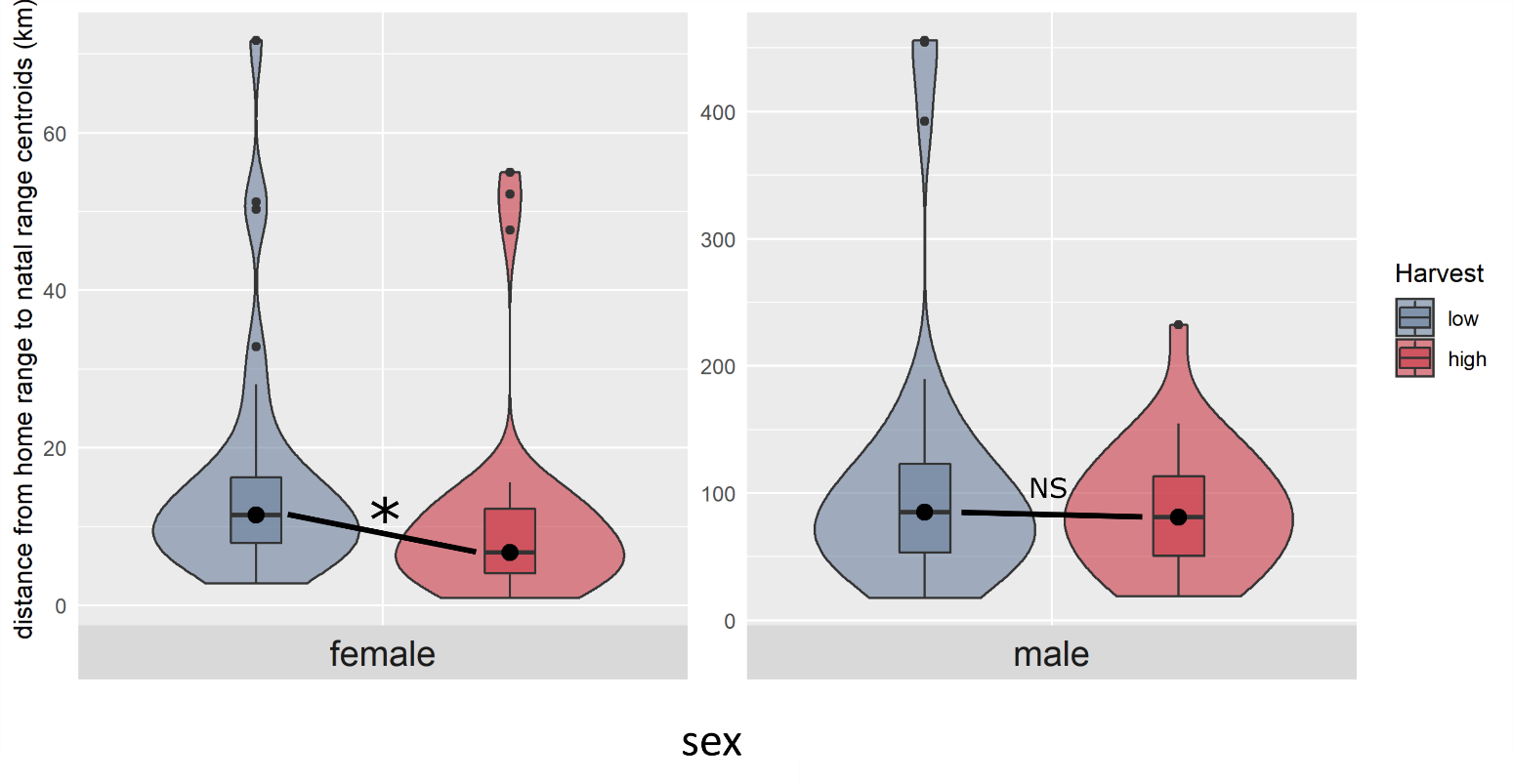


Fig. S6. The dispersal distances of female (A) and male (B) brown bears between periods of low and high harvest intensity in Sweden. Bears were assigned to low or high harvest, depending on the period they turned 4 years of age, i.e., post-dispersal. There was a significant difference (*) between low and high harvest for female distances (Mann-Whitney U test, *P* < 0.01), but not males (NS = not significant; *P* = 0.66).


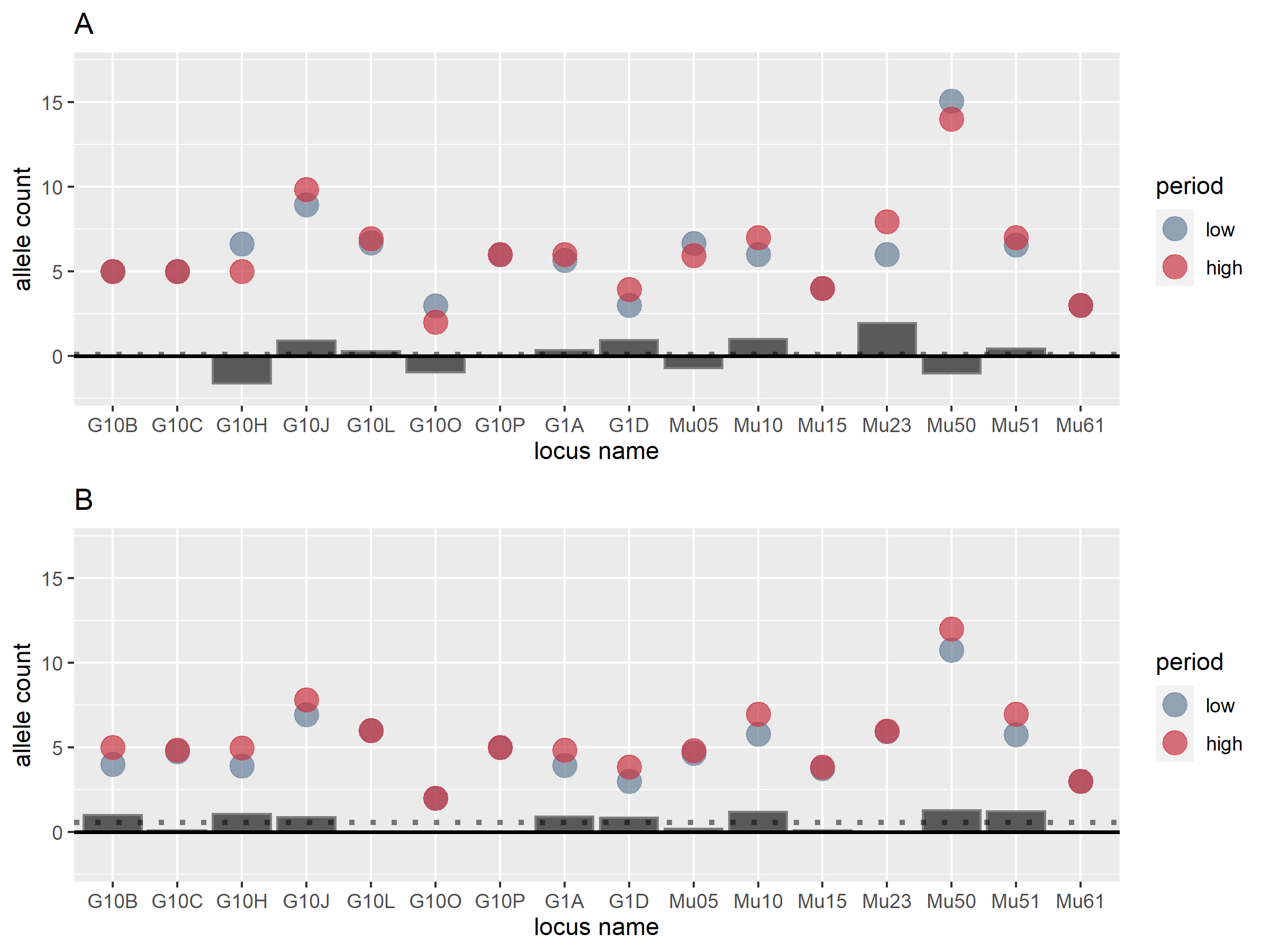


Fig. S7. Allelic richness by locus in the population (A) and matriline data sets (B) for adult female brown bears between periods of low and high harvest intensity in southcentral Sweden. Bears were assigned to low or high harvest, depending on which period they spent most of their lives. Gray and red circles mark the number of unique alleles at each locus for low and high harvest, respectively, with the dark gray bars depicting either a positive, negative, or ~no change to richness. There appeared to be a mean change (dotted horizontal line) in allelic richness for the matriline data set compared to 0 (solid horizontal black line), but not for the population data set.

Table S1. Table was adapted from (Frank et al. 2017a) and provides summary statistics for microsatellites used for pedigree reconstruction and Lynch and Ritland’s (1999) relatedness index for brown bears (N = 1614) in southcentral Sweden: N_A_ = Number of alleles; H_o_ = observed heterozygosity; H_e_ = expected heterozygosity; P_ex_ = probability of exclusion for a single unknown parent; P_id_ = probability of identity for unrelated individuals; G_e_ (%) = genotyping error rate by locus for 120 individuals that were genotyped at least twice. Averages were calculated for N_A_, H_o_, H_e_, and G_e_ whereas overall probabilities across all loci were calculated for P_ex_ and P_id_.

| Locus | N_A_ | H_o_ | H_e_ | P_ex_ | P_id_ | G_e_ |
| --- | --- | --- | --- | --- | --- | --- |
| G1A^a^ | 6 | 0.625 | 0.630 | 0.380 | 0.810 | 0.0 |
| G1D^a^ | 7 | 0.592 | 0.620 | 0.340 | 0.786 | 0.4 |
| G10B^a^ | 8 | 0.699 | 0.703 | 0.442 | 0.855 | 0.0 |
| G10C^b^ | 5 | 0.697 | 0.698 | 0.460 | 0.864 | 0.5 |
| G10J^c^ | 6 | 0.574 | 0.570 | 0.325 | 0.762 | 0.4 |
| G10H^c^ | 9 | 0.525 | 0.545 | 0.308 | 0.740 | 0.6 |
| G10L^a^ | 8 | 0.757 | 0.760 | 0.544 | 0.905 | 0.0 |
| G10O^a^ | 3 | 0.392 | 0.397 | 0.166 | 0.564 | 0.9 |
| G10P^b^ | 6 | 0.736 | 0.757 | 0.535 | 0.902 | 0.0 |
| Mu05^d^ | 8 | 0.640 | 0.636 | 0.365 | 0.797 | 0.5 |
| Mu10^d^ | 8 | 0.806 | 0.806 | 0.610 | 0.933 | 0.0 |
| Mu15^d^ | 4 | 0.658 | 0.643 | 0.364 | 0.805 | 0.0 |
| Mu23^d^ | 8 | 0.709 | 0.699 | 0.485 | 0.876 | 4.7 |
| Mu50^d^ | 10 | 0.735 | 0.756 | 0.538 | 0.903 | 1.1 |
| Mu51^d^ | 9 | 0.824 | 0.796 | 0.592 | 0.926 | 2.0 |
| Mu61^d^ | 4 | 0.529 | 0.542 | 0.272 | 0.714 | 0.0 |
| Average/Overall | 7 | 0.656 | 0.660 | >0.99 | >0.99 | 0.1 |

^a^Paetkau and Strobeck (1994); ^b^Paetkau et al. (1995); ^c^Paetkau et al. (1998); ^d^Taberlet et al. (1997).

Table S2**.** Settings for both Cervus 3.0 (parentage assignment) and COLONY (sibship reconstruction), the latter of which recovered fathers of unknown identity but held in common among offspring. Cervus analyses were performed for father only (when mothers were known) and for both parents. The output was used to construct a pedigree for brown bears in Sweden.

| **Software** | | **Father only** | **Both parents** |
| --- | --- | --- | --- |
| Cervus | | | |
|  | *Simulations* |  |  |
|  | N offspring simulated | 10,000 | 10,000 |
|  | N candidate fathers | 128 | 125 |
|  | N candidate mothers | – | 96 |
|  | % sampled fathers | 75 | 75 |
|  | % sampled mothers | – | 90 |
|  | % loci typed | 97 | 97 |
|  | % loci mistyped | 1 | 1 |
|  | Minimum loci typed | 8 | 8 |
|  | *Assignments* |  |  |
|  | N offspring | 321 | 1463 |
|  | N father assigned | 253 | 407 |
|  | N mother assigned | – | 554 |
| COLONY | | | |
|  | N offspring | 68 | – |
|  | N candidate mothers | 36 | – |
|  | Model type | Inbreeding | – |
|  | Mating system | Polygamy | – |
|  | Prob. a mother is included in the candidates | 0.95 | – |

Table S3. Summary of fine-scale genetic structure (FGS) metrics (F_ST_, G′_ST_, Jost’s D, and Nei’s G_ST_) comparing adult female brown bears (≥4 years old) between low and high harvest in southcentral Sweden and among matrilines. Two data sets were used: population data set (N = 337 females) and the matriline data set (N = 109 females successfully assigned to matrilines). Two population subdivisions were used, i.e. harvest period (N = 2; low and high) and matriline ID (N = 8 IDs), across which genetic structure was estimated. Harvest period was used as the population subdivision for both population and matriline data sets, but matriline ID was only used for the matriline data set. Permutation tests were used for all FGS metrics to test (α = 0.05) whether observed values were different than otherwise produced by random (Null = no difference). 95% confidence intervals (CIs) were calculated from bootstrapped values (N = 1000) for each FGS metric. For FGS of matrilines, each low and high harvest was calculated independently and bootstrapped distributions were compared with a two-tailed t-test (α = 0.05) to detect changes in FGS across time. Visualization of permutation tests is shown in Fig. S3.

| dataset | subdivision | harvest period | metric | FGS value | Bootstrap CI 95% | t-test: low vs high (p-value) | Permutation (p-value) |
| --- | --- | --- | --- | --- | --- | --- | --- |
| population | period | both | F_ST_ | 0.000 | -0.001 — 0.001 |  | 0.827 |
| population | period | both | G'_ST_ | 0.001 | -0.001 — 0.004 |  | 0.587 |
| population | period | both | Jost's D | 0.001 | -0.001 — 0.003 |  | 0.660 |
| population | period | both | Nei's G_ST_ | 0.000 | 0.000 — 0.001 |  | 0.617 |
| matriline | period | both | F_ST_ | 0.002 | -0.002 — 0.007 |  | 0.516 |
| matriline | period | both | G'_ST_ | 0.004 | -0.007 — 0.02 |  | 0.601 |
| matriline | period | both | Jost's D | 0.002 | -0.005 — 0.013 |  | 0.604 |
| matriline | period | both | Nei's G_ST_ | 0.001 | -0.001 — 0.003 |  | 0.617 |
| matriline | matriline | low | F_ST_ | 0.130 | 0.099 — 0.169 |  | <0.001 |
| matriline | matriline | high | F_ST_ | 0.080 | 0.057 — 0.097 | <0.001 | <0.002 |
| matriline | matriline | low | G'_ST_ | 0.250 | 0.156 — 0.345 |  | <0.003 |
| matriline | matriline | high | G'_ST_ | 0.180 | 0.132 — 0.238 | <0.001 | <0.004 |
| matriline | matriline | low | Jost's D | 0.160 | 0.099 — 0.225 |  | <0.005 |
| matriline | matriline | high | Jost's D | 0.120 | 0.083 — 0.167 | <0.001 | <0.006 |
| matriline | matriline | low | Nei's G_ST_ | 0.100 | 0.056 — 0.144 |  | <0.007 |
| matriline | matriline | high | Nei's G_ST_ | 0.050 | 0.045 — 0.077 | <0.001 | <0.008 |
